# Epigenomics of Major Depressive Disorders and Schizophrenia: Early Life Decides

**DOI:** 10.3390/ijms18081711

**Published:** 2017-08-04

**Authors:** Anke Hoffmann, Vincenza Sportelli, Michael Ziller, Dietmar Spengler

**Affiliations:** Max Planck Institute of Psychiatry, Translational Psychiatry, 80804 Munich, Germany; hoffmann@psych.mpg.de (A.H.); vincenza_sportelli@psych.mpg.de (V.S.); michael_ziller@psych.mpg.de (M.Z.)

**Keywords:** epigenomics, early life adversity, major depression, schizophrenia, GWAS, EWAS, meQTL, eQTL

## Abstract

Brain development is guided by the interactions between the genetic blueprint and the environment. Epigenetic mechanisms, especially DNA methylation, can mediate these interactions and may also trigger long-lasting adaptations in developmental programs that increase the risk of major depressive disorders (MDD) and schizophrenia (SCZ). Early life adversity is a major risk factor for MDD/SCZ and can trigger persistent genome-wide changes in DNA methylation at genes important to early, but also to mature, brain function, including neural proliferation, differentiation, and synaptic plasticity, among others. Moreover, genetic variations controlling dynamic DNA methylation in early life are thought to influence later epigenomic changes in SCZ. This finding corroborates the high genetic load and a neurodevelopmental origin of SCZ and shows that epigenetic responses to the environment are, at least in part, genetically controlled. Interestingly, genetic variants influencing DNA methylation are also enriched in risk variants from genome-wide association studies (GWAS) on SCZ supporting a role in neurodevelopment. Overall, epigenomic responses to early life adversity appear to be controlled to different degrees by genetics in MDD/SCZ, even though the potential reversibility of epigenomic processes may offer new hope for timely therapeutic interventions in MDD/SCZ.

## 1. Introduction

The burden of major depressive disorders (MDD) and schizophrenia (SCZ) is on the rise globally, reflecting ongoing population aging and demographic growth. In 2015, ≈300 million people were affected by MDD; this corresponds to 4.4% of the global population and an increase by 18% across the last 10 years [1]. At the same time, ≈21 million were affected by SCZ [2]. For comparison, ≈35 million people were affected by cancer.

MDD is the leading cause of life with disability and is a major contributor to the overall burden of disease [3,4]. More women are affected by depression than men [1]. In contrast to usual mood fluctuations in daily life, MDD is a serious health condition characterized by a depressed mood, loss of interest and enjoyment, and reduced energy, leading together to poor function at work, at school, and in the family [5,6]. Additional symptoms are feelings of guilt or low self-worth, anxiety, disturbed appetite and sleep, poor concentration, and even unexplained physical symptoms. Depressive episodes can last over an extended period of time or manifest remission and recurrent relapses [6]. At its worst, depressed people commit suicide, with close to 800,000 victims every year. Therefore, MDD represents the second leading cause of death in 15 to 19 year olds [1].

SCZ is not as common as MDD but is more common among males (12 million) than females (nine million) and also develops earlier among men [2]. Distortions in perception, thinking, language, emotions, sense of self, and behavior are characteristic for SCZ and give rise to the ample delusions that can associate with acoustical (hearing voices), optical, and sensory hallucinations [6]. Behavioral abnormalities include self-neglect, wandering aimlessly, strange appearance, incoherent speech, mumbling, or laughing to oneself. SCZ causes great disability that may interfere with educational and occupational performance. Overall, individuals with SCZ are 2 to 2.5 times more likely to die early than the general population due to cardiovascular, metabolic, and infectious diseases [2].

Research on MDD/SCZ has not identified a single causal factor. Instead, psychiatric diseases are hypothesized to result from complex interactions of social, psychological, and biological factors during neurodevelopment and beyond. The neurodevelopmental hypothesis of psychiatric diseases dates back to the latter part of the nineteenth century, when authorities such as Clouston [7] posited that at least some insanities were ‘developmental’ in origin. With the spread of Kraepelin’s concept of dementia praecox as a degenerative disease (see pp. 426–441 [8]), this view passed largely into oblivion until the 1980s when several research groups again began to speculate that SCZ might have a significant neurodevelopmental component [9,10,11]. Likewise, more than 100 years have passed since Sigmund Freud [12] postulated the important role of early traumatic experiences on the development of depression and major disorders. Still, it was not before the late 1980s that the critical role of early life events and parenting for the development of MDD was re-addressed at the biological scale [13,14,15,16].

With respect to the neurodevelopmental origin of SCZ/MDD, epigenetic mechanisms (see below) can serve as an interface between the genetic blueprint and the environment and were originally recognized to mediate the interactions between intrinsic and extrinsic clues during cellular and organismal development [17]. More recently, epigenetic mechanisms have been hypothesized to mediate also between genes and social experiences in translational rodent studies as well as in men [18,19]. Especially, experiences during early life can influence lastingly the expression of genes controlling neuronal cell numbers, neuronal activity, and connectivity through epigenetic mechanisms; all of these processes are well-known to regulate behavior, cognition, and mood, among other higher brain functions. While studies on gene-environment interactions have centered mostly on single genes known to act in pathways that are thought to be involved in mental diseases [20,21], recent genome-wide approaches have provided new insights into epigenomic changes in MDD/SCZ and their potential interaction with genetic variation.

Here, we refer to previous discoveries on the genetic architecture of MDD/SCZ and the emerging role of epigenomic studies (Section 2). We also evaluate the eminent role of early life for future mental health (Section 3) and recent insights into the dynamic role of DNA methylation during early brain development (Section 4). In this context, we explore the hypothesis that early life experience, particularly adversity, can lead to enduring epigenetic changes that increase the risk of later MDD/SCZ. With respect to MDD, findings on epigenetic changes in rodent models and postmortem human brains will be discussed (Section 5). Further, we will consider the latest results on epigenetic changes in SCZ and how they intersect with genetic risk variants (Section 6). Concluding, we will discuss future steps to be taken to address current limitations and to advance insight into the functional implications from these findings by using human pluripotent stem cell models.

## 2. The Genetic Architecture of MDD and SCZ

The advent of high-throughput genotyping technologies has pioneered insight into common genetic variation contributing to psychiatric diseases. More than 85 million single nucleotide polymorphisms (SNPs) have been identified in the human population, accounting for 95% of all known sequence variants [22]. For practical reasons, genome-wide association studies (GWAS) include only a few tag SNPs that represent all SNPs in the same linkage disequilibrium (LD) block. Multiple SNP associations within the same LD block are considered to detect a single causal variant, the physical boundaries of which are statistically inferred for the identified SNP associations. Since tag SNPs capture all the other SNPs localizing to the risk-associated haplotype block, they are unlikely to encode on their own the causal genetic variant that underlies the disease association. 

Family history is a strong and well-replicated risk factor that associates with different heritability estimates for MDD (0.37) and SCZ (0.81) [23,24,25]. In the case of MDD, however, the combination of high prevalence and moderate heritability poses a major challenge to the genetic analysis of MDD. Consistent with this concern, a seminal study four years ago on more than 70,000 MDD cases and controls from combined data sets did not obtain any evidence for any variant significant at genome-wide association thresholds [26].

Likewise, people manifesting the same MDD or SCZ symptoms may not share the same etiology, and no robust laboratory tests exist to distinguish between subtypes within either condition. Until now, neuroscience and genetic studies into psychiatric disorders rely generally on disease definitions that are based on the influential “Diagnostic” and Statistical Manual of Mental Disorders; (DSM) that was designed as a purely diagnostic tool [27]. Although DSM considers different disorders as distinct entities, the boundaries between disorders are often not as strict as the DSM suggests. To develop an alternative framework for research into psychiatric disorders, the US National Institute of Mental Health (NIMH) introduced in 2009 its Research Domain Criteria (RDoC) project to develop a research classification system for mental disorders based upon dimensions of neurobiology and observable behavior [28]. RDoC supports research to explicate fundamental biobehavioral dimensions that cut across current heterogeneous disorder categories, with the goal to transform the approach to the nosology of mental disorders. While progress in the diagnostic validity of major psychiatric disorders is thus still looked for, a number of more recent studies have sought to advance insight into the genetics of psychiatric disorders by increasing case control numbers or by narrowing the range of clinical phenotypes. 

First, the CONVERGE consortium [29] minimized genetic heterogeneity by including only women of Han Chinese ethnicity, among whom depression is thought to be under-diagnosed and among whom those who were diagnosed with two or more episodes of MDD are likely to represent more severe forms of the disease. Two regions in which genetic variants associate with MDD were identified; one mapping near *SIRT1* (encoding Sirtuin 1, an NAD(+)-dependent histone deacetylase) and the other mapping in an intron of *LHPP* (encoding a protein phosphatase that cleaves phospholysine and/or phosphohistidine bonds). These variants were corroborated with a second method and in an independent sample, suggesting that the proximity of one of the variants to *SIRT1* could contribute to deregulated mitochondrial energy metabolism [30]. Since these two risk allele variants are extremely rare in Europe [26], they may be actually restricted to severe cases of MDD from China. 

Second, Hyde et al. [31] dramatically increased the cohort size (including 75,607 cases with self-reported or clinical diagnosis or treatment for depression and 231,747 controls) and performed meta-analysis of these data with published MDD genome-wide association study results. This approach identified 17 MDD-associated variants in 15 regions of the genome in people of European descent. While the question arises as to how well self-reports correspond to the kind of depression physicians see in the clinic [32], evidence for shared polygenic risk between their MDD cases and published SCZ cases [33] provides initial support for this phenotyping approach.

Third, Power et al. [34] reanalyzed data from a previously published meta-analysis conducted by the Psychiatric Genomics Consortium (PGC) [26] by acknowledging that earlier onsets of MDD show greater familial loading [35,36]. Discovery case-control studies (8920 cases and 9521 controls) were stratified using increasing/decreasing age-at-onset cutoffs and led to the identification of one replicated genome-wide significant locus with adult-onset MDD that had not reached significance in the original unstratified PGC meta-analysis. Interestingly, polygenic score analysis additionally showed that earlier-onset cases of MDD share a greater genetic overlap with SCZ and bipolar disorder (BIP) than adult-onset cases.

Contrary to MDD, higher heritability in SCZ enabled the identification of common variants encoding subtle effects as well as rare but highly penetrant copy number variations and possibly exome variants [25]. Ripke et al. [37] recently estimated that 6333 to 10,200 independent and mostly common SNPs may underlie the risk for SCZ, with each conferring a small increase in risk. Incrementally, these SNPs are thought to account for around 50% of the total variance in liability to SCZ and indicate that common genetic variation is a major factor in SCZ heritability. Since the first GWAS for SCZ was published in 2009 [38,39], the size of the studies and the number of loci associated with the condition has expanded, whereby the latest study [40] included more than 150,000 people and identified 108 genomic regions containing genetic risk factors. Since the identified risk variants are common, they will contribute to most, if not all, cases. The authors estimate that the 108 loci (hereafter referred to as Psychiatric Genomics Consortium (PGC) risk variants) collectively implicate a total of 305 genes, including “plausible” candidates such as *DRD2* (a well-known dopamine receptor gene), a locus on chromosome 6 that harbors the major histocompatibility complex, and several genes encoding calcium channel subunits and proteins involved in synaptic plasticity. Importantly though, further studies are needed to map the genetic variation-to-genes-to-function to corroborate the role of these and others candidates in SCZ.

Collectively, genetics is a major cause of MDD/SCZ. Despite considerable progress on the genetic architecture of SCZ, causal variants from GWAS still remain to be verified. Presently, a substantial fraction of the risk of MDD/SCZ still remains unexplained and points to the role of environmental factors.

## 3. Early Life Events Preset Adult Behavior

From conception onwards, the physical and social environment acts on the genetic blueprint to adjust development and lifelong programs of somatic and mental functions. These interactions are particularly important during early life and can elicit long-lasting “anticipatory” changes in phenotype, referred to as “programming” [41,42]. Epigenetic mechanisms can mediate the effects of early life through sustained changes in (neuronal) gene expression that prepare the organism to effectively cope with changing environments. However, early adaptations may also result in inefficient responses due to inherent (genetic) constrains or misguided adjustments that can increase the risk of future disease [19,43].

The embryonic and early postnatal brain is highly plastic due to extensive cell proliferation that passes over to progressive differentiation [44]. For example, the embryonic human brain produces some 250,000 new cells per minute [45] and forms about 40,000 synapses per minute in the last trimester [46]. Early-born neurons dynamically organize themselves into functional networks and undergo extensive pruning and reorganization from early postnatal life through early adulthood [16]. Various environmental cues and life events can act on these cellular processes and trigger lasting (epigenetic) changes in gene regulatory networks. 

Adverse events, especially in early life, are the greatest risk factor for the development of MDD [47]. Early adversity can comprise interpersonal loss (e.g., parental death), parental maladjustment (e.g., mental illness or substance abuse), low socioeconomic status in childhood, and maltreatment (physical or sexual abuse and neglect), among other threats [48]. Maltreatment is the leading cause of early life adversity in Westernized societies (prevalence 0.9%), with 80% of the cases that associate with the highest rates of increased risk for MDD corresponding to neglect [49]. Recurrent episodes of early adversity increase the risk for depression four-fold, whereby the severity of exposure correlates with the risk of life-long recurrent depression [50] and completed suicide [49]. This disease process suggests that the initial event leads to sustained changes in gene regulatory and/or neuronal networks that can be reactivated with new exposure and thereby facilitate disease development. At the same time, early life adversity is also an important risk factor for the development of SCZ [24,51,52,53]. A growing body of evidence based on epidemiological and translational studies further shows that prenatal adversity, in the form of stress and trauma, mood and anxiety disorders, or infections and severe physical illness in the mother, is a shared risk factor for MDD and SCZ [54,55,56].

A common response to early life adversity is the activation of the hypothalamic-pituitary-adrenal axis (HPA axis), a major mediator of the stress response [57] and the subsequent secretion of glucocorticoids (GC) that normally serve to restore physiological and behavioral homeostasis [58]. The action of GCs is mediated by binding to high- and low-affinity glucocorticoid receptors (MR and GR, respectively) that operate as nuclear transcription factors and through membrane bound mechanisms [59]. However, the adaptation-promoting action of GCs can turn into the opposite in case the type, strength, or duration of the stressor overwhelms the regulatory mechanisms that act to restrain GC secretion [58,59].

Taken together, early-life adversity is a major risk factor for MDD/SCZ and frequently leads to sustained deregulation of the HPA axis. Such deregulation, in turn, associates with structural brain and epigenetic changes within individual cells that can confer an increased risk of psychiatric disease.

## 4. From Epigenetics to Epigenomics

Major epigenetic mechanisms [60] comprise DNA methylation, posttranslational modifications of core histones, nucleosome positioning, and non-coding RNA (ncRNA). All of these mechanisms are thought to act jointly in “the structural adaptation of chromosomes so as to register, signal, or perpetuate activity states” (see p. 398 in [61]). Historically, DNA methylation has been the most studied and is of particular relevance to this review.

**Figure 1:**
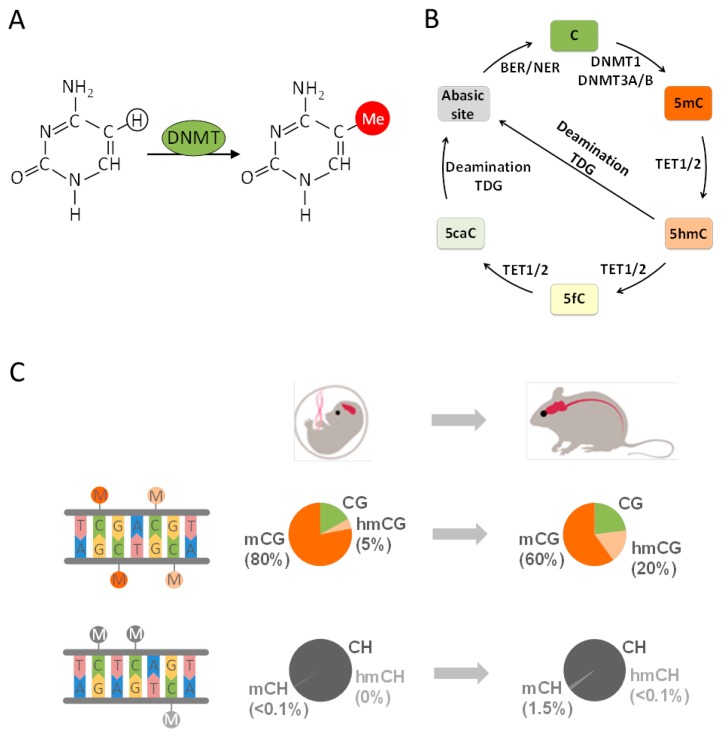
The life-cycle and distribution of DNA methylation in mammalian cells. (**A**) The nucleotide cytosine (**C**) is methylated at the 5th carbon either by de novo DNA methyltransferases DNMT3A or DNMT3B or by the DNA maintenance methyltransferase 1 (DNMT1) during DNA replication; (**B**) active demethylation of 5-methylcytosine (5mC) takes place through iterative oxidation by ten-eleven translocation proteins (TET1/2), producing 5-hydroxymethylcytosine (5hmC). This product is further oxidized to 5-formylcytosine (5fC) and lastly 5-carboxylcytosine (5caC). By an alternative route, 5caC, but also 5hmC, are deaminated to thymine and excised by thymine DNA glycosylase (TDG). Lastly, the mismatched bases are repaired by the base excision and/or nucleotide excision repair machinery (BER/NER); (**C**) cortical methylomes in fetal and adult mice. CpG methylation and non-CpG methylation are shown. Methylcytosine and hydroxymethylcytosine at CG dinucleotides are symbolized by dark and light orange lollipops, respectively. The diagrams display the percentage of unmethylated (CG or CH), methylated (mCG or mCH), and hydroxymethylated (hmCG or hmCH) cytosines. The amount of hydroxymethylcytosine rises with age at the expense of methylcytosine, indicative of a ten-eleven translocation enzyme catalyzed conversion of methylcytosine into hydroxymethylcytosine; mCH is weakly present at fetal stages but strongly rises during mice development.

Canonical DNA methylation (mCG) refers to the transfer of a methyl group to a cytosine-guanine dinucleotide (CG) (Figure 1A) and is broadly distributed across the genome. In the human brain ≈80% of all CGs are methylated similarly to other tissues [62]. mCG exists in the neural and glial cells of all brain tissues from prenatal life to old age [63] and is thought to fulfill an important role in cell differentiation and cellular identity [17]. The de novo DNA methyltransferases (DNMTs) DNMT3A and DNMT3B catalyze mCG, which is reestablished by DNMT1 following genome replication (Figure 1B). Both DNMT1 and DNMT3A are also critically involved in neuronal plasticity, learning, and memory through their joint role in DNA methylation and its effect on neuronal gene expression [64].

Mammalian genomes are commonly depleted of CG residues, except for enrichment in so-called CG islands (CGIs) that occur in less than half of all human gene promotors and remain usually methylation-free. While only few promoter CGIs undergo DNA methylation [65,66], DNA methylation is commonly found in CGI-free promoters modulating gene expression in undifferentiated and differentiated cells [67]. Historically, DNA methylation is thought to confer lasting, or even irreversible, gene repression during development and beyond. More recent reports suggest, however, a broader role in enhancing transcription through the inhibition of spurious transcription initiation or the promotion of prolongation efficiency [65]. In any case, the overall effects of DNA methylation depend critically on the genomic position, primary sequence, and pre-existing transcriptional activity.

Rapid progress in high-throughput sequencing has provided an unprecedented genome-wide view on DNA methylation by charting exact sites and sequence contexts but has also evidenced unique features of the human and mice brain methylome when compared to the respective peripheral tissues. 

First, the brain methylome contains a high amount of 5-hydroxymethylcytosine (5hmCG), which gradually accumulates during mouse development (Figure 1C) [68,69,70]. This modification is catalyzed by the family of ten-eleven translocation enzymes (TET) that oxidize mCG to 5hmCG and further derivatives (Figure 1B). The final product 5-carboxylcytosine serves as a substrate for DNA glycosylase-mediated-base excision and replacement by unmodified cytosine through the base excision and/or nucleotide excision repair machinery (BER/NER) [71]. Recent findings indicate that, in mature neurons, 5hmCG occurs as a transient modification that is replaced in a temporally and spatially confined manner in small regulatory regions of the genome [72,73,74,75].

Second, methylation in a CH context (mCH, where H corresponds to A, C, or T) is found in neuronal cells as well as in embryonic stem cells [63,76,77,78] but rarely in peripheral differentiated tissues [79,80,81]. This so-called non-canonical DNA methylation is barely detectable in the fetal and early-infant brain methylome but also rapidly accumulates postnatally across two years (Figure 1C). Thereafter, mCH gradually reaches the level of mCG by adulthood and accounts then for more than half of all neuronal methylcytosines. Since the absolute number of C residues exceeds by far the absolute number of CG dinucleotides, mCH still remains a relatively rare event in the neural methylome. Notwithstanding this reservation, mCH has been hypothesized to associate with the rapid rise in postnatal synaptogenesis and synaptic pruning that shapes neural network formation. However, a role as substrate for epigenomic changes in response to early life events has not been reported so far.

In view of an environmental causation of MDD/SCZ, dynamic changes in DNA methylation (Figure 1B,C) offer an intriguing interface for the interaction of societal risk factors with the genome. Experience driven neuronal activity connects to various transcriptional regulators that in turn recruit the epigenetic machinery. These factors can confer local [18,19,43] and genome-wide (see Section 5 and Section 6) epigenetic changes that last beyond the initial stimulus and cause changes in the gene expression underlying various brain functions.

Epigenomics refers to the study of epigenetic mechanisms at the genome-scale [82] and enables important insights into the functional relationships of genes in health and disease through the identification of regulatory mechanisms that are sensitive to environmental and lifestyle factors. Hereby, complex phenotypes are analyzed for genetically induced epigenetic alterations and/or environmentally induced epigenetic alterations that in turn can be controlled by genetic effects. Since the epigenome is highly dynamic, the extent of interindividual phenotypic variation needs to be assessed by large-scale, systematic epigenome-wide association studies (EWAS). By approach, EWAS are equivalent to GWAS, with variation at a single CG site corresponding formally to a SNP [83]. Measurements of CpG methylation average, however, thousands of DNA copies at the tissue level and represent therefore aggregate information on the effect sizes between cases and controls, as opposed to the categorical nature of SNP information.

## 5. Early Life Adversity-Dependent Epigenomic Responses as Risk Factor for MDD

The quality of maternal care critically influences brain function through lasting effects on stress regulation, emotion, learning, and memory [84,85]. In rats, naturally occurring variations in perinatal maternal care associate with changes in offspring’s behavior and hippocampal gene expression (>900 genes) that persist into adulthood [86,87]. These changes can be reversed by cross-fostering [86], pharmacological, or dietary (methyl supplementation) treatments [88] and indicate that maternal care leads to the epigenetic programming of gene expression. In support of this hypothesis, a pioneering study by Weaver et al. [89] showed that differences in early maternal care triggered differential DNA methylation at the proximal glucocorticoid receptor gene (*NR3C1*) promoter in hippocampal cells, which was prevented by co-treatment with a histone deacetylase inhibitor.

Moving beyond single gene analysis, the researchers further analyzed gene expression, histone acetylation, and DNA methylation in a large region of chromosome 18 by customized tilling arrays [90]. Differences in maternal care were associated with both increased and decreased peaks of histone acetylation and DNA methylation over one hundred kilobase pairs, covering promoters, exons, and genes. The response to maternal care appeared to be specific since not all genes were affected, whereas differences in epigenetic marks co-clustered over large distances, which was indicative of widespread epigenetic effects on multiple genes in the same genomic region. In general, increased transcription was associated with reduced DNA methylation at upstream regulatory sites, increased exonic histone acetylation, and DNA methylation. The co-clustering of epigenetic responses was highly developed at the protocadherin gene cluster (*PCDH*), which regulates synaptic development and neuronal function [91]. Taken together, these findings indicate that large groups of functionally related genes or gene networks may be coordinately regulated by early life events.

Another line of evidence for the profound effects of stress on early brain development stems from animal studies, which showed that maternally administered synthetic GCs (e.g., dexamethasone) in late gestation can induce lasting changes in HPA-axis function and behavior in adult offspring of different species, including guinea pigs, mice, sheep, and non-human primates [92,93]. Similarly, recent studies in humans have reported an increased risk of emotional and behavioral abnormalities in children exposed to elevated glucocorticoid concentrations in utero by either antenatal dexamethasone treatment (to advance lung maturation) or maternal stress [94,95]. To obtain insight into GC effects on the brain methylome, Crudo et al. [96] investigated guinea pigs, whose fetal pattern of brain development more closely resembles that of the human [97]. Genomic DNA was purified from fetal hippocampi that were isolated immediately before the fetal plasma cortisol surge (gestational day 52, GD52) or in late gestation (GD65). In parallel, genomic DNA was isolated for 24 h or 14 days (GD52 and GD65, respectively) after the completion of a serial dexamethasone application to the mother (GD40, GD41, GD50, and GD51). All of these samples were analyzed for genome wide changes in DNA methylation using promoter tilling arrays containing ≈43,000 genes. Compared to GD52, extensive genome-wide promoter hypomethylation was detected on GD65 after the fetal cortisol surge or 24 h after the last dexamethasone application. However, 14 days after dexamethasone application, these differences did not persist and a different set of promoters became hypermethylated or hypomethylated when compared to the untreated fetus on GD65 [96].

In general, changes in DNA methylation correlated negatively with genome-wide changes in transcription following the cortisol surge (1086 genes) or dexamethasone application (1126 genes) [98]. Among the 173 genes shared between both conditions, 159 genes showed the same directional change. However, none of the dexamethasone regulated genes remained similarly different from the controls at GD65, indicating that dexamethasone triggered precocious changes in expression at GD52. 

Beyond the methylome, GCs also affected GR DNA binding. When comparing GD65 to GD52, 1245 gene promoters exhibited differential GR DNA-binding, with 627 promoters showing an increase and 618 promoters a decrease. Twenty four hours after dexamethasone treatment only 94 gene promoters showed differential GR DNA binding (58 increases versus 46 decreases), as compared to 690 promoters after 14 days (279 increases versus 411 decreases). This indicates that development and GC treatment regulate GR DNA binding largely differently, whereby the dexamethasone-induced precocious maturation of GR DNA-binding is confined to a rather small set of gene promoters [98].

Overall, these results show that the fetal cortisol surge drives the genome-wide reconfiguration of the hippocampal methylome, altered transcription, and GR DNA-binding during late hippocampal development. Immediate effects from GC application mostly anticipate changes from the fetal cortisol surge; however, in the long run they induce profound changes in developmental trajectories that may underlie in part the lasting endocrine and behavioral phenotypes associated with antenatal GC treatment. 

These results raise the important question as to whether they may also extend to men. Since experimental glucocorticoid application to a human fetus is ethically inacceptable, such studies have to rely on postmortem brain analyses of people who were exposed to different stress conditions during early life. In a hypothesis-driven approach, McGowan et al. [99] originally detected differential hippocampal *NR3C1* promoter methylation and gene expression between suicided subjects with histories of childhood abuse or severe neglect relative to controls (victims of sudden, accidental death with no history of abuse or neglect). In light of previous findings in rats [90], the researchers extended their analysis to postmortem hippocampal tissue from humans with and without a history of early life adversity (12 individuals in each group) [100]. Methylation profiles covered the genomic region from 3.25 Mb upstream to 3.25 Mb downstream of *NR3C1* at 100-bp spacing, which was compared to the homologous regions in rats that had experienced differential maternal care. Methylation profiles showed hundreds of differentially methylated regions (DMR) associated with differences in early life care that were unevenly spread across the *Nr3c1* locus in rats. In humans, 281 regions were differentially methylated between individuals without and with a history of early life adversity. Among these, 126 DMRs were hypermethylated in non-exposed individuals versus 155 hypermethylated DMRs in exposed individuals. In comparison, the rat profiles showed twice as many DMRs, possibly reflecting the more homogenous study group, of which 373 and 350 DMRs were associated with high and low maternal care, respectively. 

Moreover, DMRs showing the same direction of change in response to early life experiences clustered in large genomic regions, indicating a high level of organization connecting distant sites. For example, DMRs associated with early adversity clustered at the *PCDH* locus in both species. Taken together, these findings suggest that epigenomic responses to early life adversity are conserved between rats and humans and can affect broad regions in the hippocampus, including the *NR3C1* and *PCDH* loci.

Following this, the research teams [101] extended their investigations to the genome-wide analysis of DNA methylation in individuals (*n* = 25) exposed to early life adversity (i.e., severe abuse during childhood) in comparison to non-exposed controls (*n* = 16). Neuronal and non-neuronal cells from hippocampal tissues were sorted by fluorescence-assisted cell sorting, and methylated DNA fractions were isolated by immunoprecipitation and subsequently hybridized to a custom-designed 400K promoter tilling array containing 23,551 proximal promoter regions. The methylation profiles were also compared with the corresponding genome-wide expression profiles derived from RNA microarrays. This approach led to the identification of 362 differentially methylated promoters in individuals exposed to early abuse when compared to controls. Among these promoters, 248 were hypermethylated and 114 hypomethylated, whereby methylation differences occurred mostly in the neuronal cell fraction. Abuse associated epigenetic alterations were evenly distributed throughout the genome, and most of the methylation changes correlated inversely with gene expression levels. Functional annotation clustering analysis evidenced enrichment in genes associated with neuronal plasticity, including cell adhesion and signaling. For example, the most differentially methylated gene corresponded to *ALS2* (Alsin), a member of the guanine nucleotide exchange factors for the small GTPase RAB5 (RAS-associated protein), which plays a crucial role in intracellular endosomal trafficking.

In a further study, the researchers also analyzed genome-wide methylation changes in the hippocampus of suicide completers (*n* = 46 subjects) versus non-psychiatric sudden-death subjects (*n* = 16) by means of their customized 400K tilling array [102]. Predisposition to suicide partially overlaps with vulnerability to depression and strongly associates with depressive psychopathology [103]. Similar to depression, suicide results from the interactions between the genetic, developmental, and social risk factors [104] that are thought to act through lasting mechanisms on brain function. In order to assess the role of epigenomic changes in suicide, Labonté et al. [102] performed an analysis of methylated DNA fractions from neuronal and non-neuronal hippocampal cells, as described above. Additionally, the effects from epigenomic changes on gene expression were assessed by expression profiling on a substantial subgroup of the same tissue samples. The researchers detected 366 differentially methylated promoters in suicide completers relative to comparison subjects, which were evenly distributed across the genome. Among these, 273 promoters were hypermethylated and 93 promoters were hypomethylated, whereby DNA methylation anti-correlated in general with gene expression differences. Functionally, DMRs were enriched in promoters of genes involved in behavioral and cognitive processes, including learning, memory, and synaptic transmission (e.g., *CHRNB2*, encoding neuronal acetylcholine receptor subunit beta-2; *GRM7*, encoding metabotropic glutamatergic receptor 7; and *DHB*, encoding dopamine beta-hydroxylase), which have been associated with vulnerability to suicide [105].

In sum, these results suggest an important role for epigenomic changes in genes regulating behavioral and cognitive processes in the hippocampus of suicide completers. While these findings resemble those on epigenomic effects in response to early life adversity, the DMRs identified in the two studies were different and are known to affect different pathways. Hence, epigenomic changes due to early life adversity seem to recapitulate specific early events rather than a general response to near-term psychopathology. If this is the case, further studies on genome wide epigenomic changes in MDD associated with early life adversity are looked for to deepen our understanding of the molecular pathways affected. It is important to caution, however, that future studies need also to address the cell type specificity of epigenetic effects [106] in face of the high diversity of neuronal cell types in individual brain regions, which can dilute epigenetic marks in bulk tissue preparations and confound any analysis of pathway specific effects. This issue is particularly relevant for the assessment of differentially methylated regions and CpGs across brain development and in disease states in which cell type composition is known to change, possibly due to the disease condition. Such changes include the transition from mitotically active cells to differentiated cells, adjustments in developmental trajectories in response to various environmental insults, and the immigration of immune cells in case of inflammatory and neurodegenerative processes, among other events. Thus, it is critical to properly control for potential differences in cell type composition between cases and controls by e.g., purifying specific populations. 

## 6. Epigenomics in SCZ

Over the last years, an increasing number of studies have investigated epigenomic changes in rodent brains treated with neuroleptics or in peripheral tissues in medicated and non-medicated schizophrenic patients [107,108,109]. Here, we will focus on recent reports that examined epigenomic changes in postmortem brain tissues from people diagnosed SCZ and will discuss how these findings relate to early brain development and genetic variation.

### 6.1. Epigenomic Changes in SCZ Are Enriched at Neurodevelopmental Loci

The first methylome analysis of postmortem frontal brain tissues from individuals diagnosed with SCZ (*n* = 35) or bipoloar disorder (*n* = 35) and from matched controls (*n* = 35) was carried out by Mill et al. [110] by hybridization of enriched unmethylated DNA fractions to CGI-arrays. This analysis showed disease-associated DNA methylation differences in multiple loci, particularly in genes regulating glutamatergic and GABAergic neurotransmission, which are thought to be dysregulated in SCZ. Epigenetic dysregulation also affected genes involved in neuronal development (e.g., *WNT1*, encoding a secreted glycoprotein), learning and motor functions (e.g., *LMX1* and *LHX5*, encoding homeobox transcription factors). The researches further assessed the number of connections between nodes representing correlated methylation observed between different genomic loci. This network approach suggested a lower connectivity of epigenomic changes in major psychosis, pointing to a systemic epigenetic dysfunction in SCZ. 

A more recent study on genome-wide DNA methylation changes in postmortem frontal cortex from 24 patients with SCZ and 24 unaffected controls used the Illumina Infinium HumanMethylation450 Bead Chip array, containing 485,000 CpG sites (hereafter referred to as 450K array) [111]. Among these, 4641 probes corresponding to 2926 unique genes were differentially methylated, including *NOS1* (encoding neuronal nitric oxidase 1), *AKT1* (encoding protein kinase B involved in neuronal proliferation, survival, and differentiation), *DNMT1*, and *SOX10* (encoding a transcription factor from neural crest development), among other genes previously associated with SCZ and neurodevelopment. Since the patients diagnosed SCZ were not medication-free, these results raise the question of drug effects [109] and point to the need for longitudinal studies on medication free subjects.

The necessity for longitudinal studies to evaluate epigenomic changes in SCZ, was firstly approached by Pidsley et al. [112], who investigated epigenomic changes in postmortem prefrontal cortex (PFC) and cerebellum from 20 schizophrenic patients and 23 matched controls by the 450K array and subsequently assessed the disease-associated regions in human fetal cortex samples, spanning 23 to 184 days post-conception. Highly significant differentially methylated CpGs were detected in the PFC; specifically, probes in four genes were associated with SCZ at a false discovery rate (FDR) ≤ 0.05: *GSDMD*, promoting programmed necrosis that occurs upon the activation of inflammatory caspases; *RASA3*, encoding GTPase-activating protein-1; *HTR5A*, a human serotonin receptor subtype, and *PPFIA1*, a tyrosine-phosphatase interacting with protein regulation neuronal arborization, spine, and synapse numbers.

The correlation of DNA methylation across adjacent sites [113,114] allows us to aggregate single CpGs in regions and thereby to reduce complexity in the comparison of large sample sizes. This approach corroborated SCZ-associated DNA methylation differences and evidenced additionally hypomethylation in *Neuritin 1* (*NRN1*) that plays a well-established role in neurodevelopment, synaptic plasticity, and the prevention of the effects from chronic stress [115,116].

In order to establish a system-level view of DNA methylation differences associated with SCZ, the researchers carried out additionally a weighted gene co-methylation network analysis [117]. This approach led to the identification of 100 prefrontal modules, with each representing discrete networks of co-methylated sites. Such SCZ-associated co-methylated modules were enriched in neurodevelopmental pathways and loci previously implicated in SCZ. On the other hand, no SCZ-associated modules were identified in the cerebellum, which was consistent with region-specific DNA methylation differences in SCZ. 

In further support of these findings, SCZ-associated differentially methylated CpGs were enriched for CpGs that underwent dynamic methylation changes during fetal neocortical development. Specifically, 44% of SCZ-associated CpGs were associated with post-conception age in the developing fetal brain indicating a highly significant enrichment for neurodevelopmental differentially methylated CpGs. 

Taken together, this study identified discrete modules of co-methylated loci in the PFC associated with SCZ that are significantly enriched for genes guiding neurodevelopment. Additionally, the methylomic profiling of fetal cortices evidenced that SCZ-associated differentially methylated CpGs undergo dynamic changes in DNA methylation during development. Conclusively, these data strengthen a neurodevelopmental component in SCZ with epigenetic mechanisms, possibly mediating between neurodevelopment dysregulation and risk of disease.

### 6.2. Epigenomic Changes in SCZ Are Genetically Controlled

Epigenetic mechanisms can act as an interface between a genetic blueprint and environmental exposures/developmental cues and are on their own controlled by genetics. In light of SCZ’s high heritability, a line of recent studies has examined the impact of genetics on epigenomic changes in SCZ.

**Figure 2:**
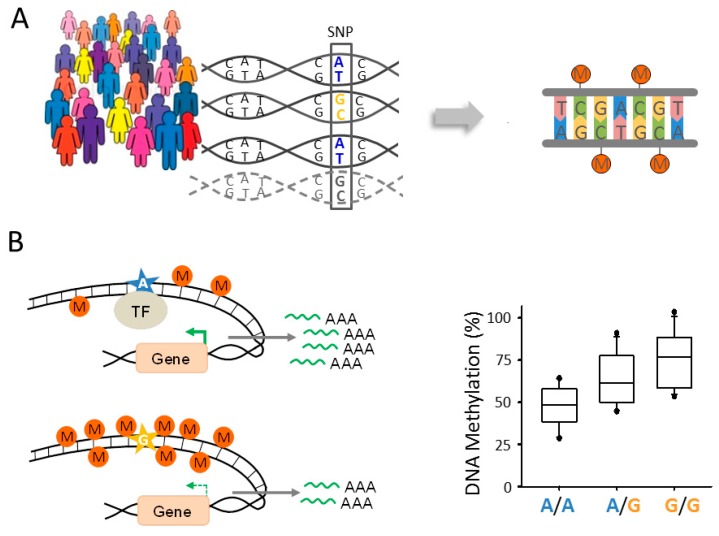
Role of genetic variation for DNA methylation. (**A**) Single nucleotide polymorphisms (SNPs) can control levels of DNA methylation in a population. In this example, the presence of the nucleotide base guanine (G; marked in light orange) is thought to promote DNA methylation at unrelated distant sites (right). The opposite outcome is hypothesized for the nucleotide adenine (A; marked in blue). The dashed line symbolizes further examples, which altogether are used to infer the allele frequency of the respective SNP within the population. In general, genetically induced changes in DNA methylation critically depend on the developmental stage and environmental context to affect gene expression; (**B**) model for *cis*-meQTLs. A local *cis*-acting SNP variant (A-allele marked by a blue star) maps to a regulatory element; for example, a transcription factor (TF) binding site. Sequence variation (G-allele marked by a light orange star) can lead to a reduced binding of the TF, decreased gene transcription (symbolized by green arrows of different strength when comparing the A- to the B-allele), and encroachment of DNA methylation (M), symbolized by filled dark orange circles. As shown by the chart, *cis*-meQTLs can lead to differences in the amount of CpG-methylation between two copies of an allele. Homozygous carriers of the transcriptional active A-allele show less DNA methylation when compared to homozygous carries of the transcriptional less active G-allele or heterozygous carriers (right).

The conceptual groundwork for these studies was established in 2001, when Jansen and Nap [118] first proposed the term “genetical genomics” for the identification of genes regulated by genetic variation. Similar to other quantitative trait loci (QTL) that can influence any given trait of interest (e.g., growth, body weight, and disease risk), expression or methylation QTLs (eQTLs and meQTLs, respectively) are identified by measuring gene expression or DNA methylation in panels of genetically different, genotyped people (Figure 2A). Statistical association tests are used to compare expression or methylation levels with the respective genotype of each subject in order to infer eQTLs and meQTLs, respectively. A meQTL is therefore defined as a genomic region that contains one or more DNA sequence variants that regulate the methylation level of other DNA regions harboring regulatory or genic sequences or sequences of unknown functions [119]. Genetically controlled changes in DNA methylation can, but do not necessarily, translate into persistent changes in gene expression. For example, meQTLs may act only in a spatiotemporal or context dependent manner by regulating transcription during sensitive developmental time windows or in the presence of neuronal activation. 

Formally, eQTLs and meQTLs are distinguished according to their relative location to the affected genic or non-genic region(s). Local QTLs reside near the site(s) that they regulate and occur to a similar degree (10,000–20,000 QTLs) in human peripheral [120,121,122,123] and brain [124,125,126] tissues. The allele encoding the QTL operates in *cis* by regulating the copy of the gene that localizes on the same physical chromosome (Figure 2B). Frequently, *cis*-eQTLs encode allele-specific differences in regulatory DNA elements, e.g., SNPs in the DNA-binding site of a transcription factor that lead to changes in gene expression and DNA methylation. Both expression and methylation *cis*-QTLs are in general of large effect size and can be identified in fewer than one hundred samples [120,127,128,129].

Alternatively, QTLs can operate in *trans* through altering the expression, structure, or function of a diffusible factor [130]. *Trans*-QTLs are of smaller effect size and do not show the allele-specific differences in gene expression or DNA methylation that are typical for *cis*-QTLs. 

Both kinds of QTLs can also reside further away from the gene(s) they control. Traditionally, such distant QTLs were thought to operate in *trans*, a view that needs, however, to be reconsidered given the highly dynamic and topologically structured nucleus [131]. 

The role of meQTLs in psychiatric diseases was firstly addressed by Gamazon et al. [132] through the analysis of postmortem brain tissues from people diagnosed with BIP (bipolar disorder), a condition sharing substantial genetic overlap with SCZ [33]. Initially, the researchers re-assessed previously published meQTL data from 153 cerebella collected from BIP and control individuals [133] by the inclusion of imputed genotype data. This analysis detected 5974 different genes associated with a *cis*-meQTL. Further, they found that genetic variants regulating DNA methylation levels are enriched in top-ranking risk variants from BIP GWAS. Specifically, 132 *cis*-meQTLs fulfilled these conditions and matched ≈14% of the most significant associations from two previous BIP GWAS. About half of these *cis*-meQTLs corresponded additionally to a *cis*-eQTL, raising the possibility that BIP risk variants jointly regulate DNA methylation and gene expression. However, only a few SNPs among those that were most significantly associated with BIP seemed actually to control both DNA methylation and gene expression (hereafter referred to as combined SNPs) in postmortem brain tissues. This result is in accord with previous reports [120,124,134,135], indicating that the effects of a substantial proportion of meQTLs on gene expression may be context-dependent (see above). Noteworthy, one combined BIP-associated SNP localized in *DLG5*, encoding a scaffolding protein that regulates precursor cell division and proliferation, epithelial cell polarity, cell migration, and adhesion; all of these processes are key to early neural stem cells and neurodevelopment [136].

Following this, Numata et al. [137] conducted a comprehensive meQTL analysis on dorsolateral prefrontal cortices (DLPFC) collected from 106 individuals diagnosed with SCZ and 110 matched controls. Although the researches still used the Illumina 27K array (containing 27,578 CpG sites spanning 14,495 genes), they detected in their *cis*-analysis (*cis* identified as within 1 Mb of a CpG site) that 36,366 SNP-CpG pairs were significantly correlated, independent from the case-control status, and corresponded to 18,452 *cis*-meQTLs.

Taken together, these studies corroborate the existence of abundant meQTLs in the human brain and indicate that epigenomic changes in SCZ are at least in part genetically controlled by GWAS risk variants.

### 6.3. A Role for Fetal meQTLs in Mediating the Genetic Risk for SCZ

Extending the above findings further, two recent landmark studies on SCZ have comprehensively studied the role of CpG methylation and meQTLs in human fetal and adult brains and how they intersect with genetic risk variants from SCZ GWAS [125,126].

**Figure 3:**
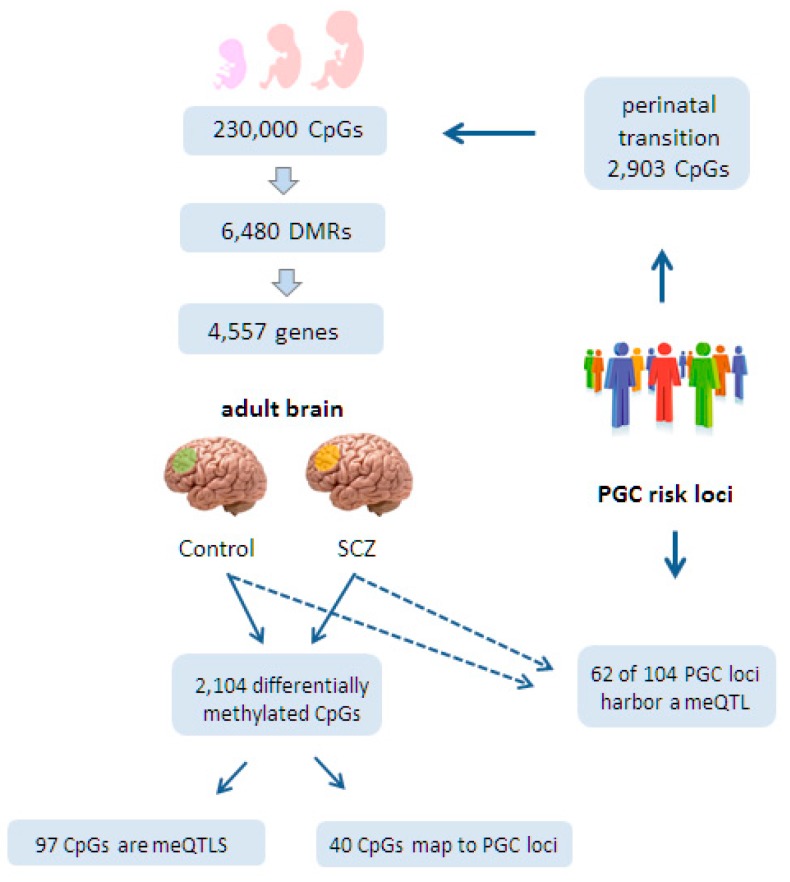
CpG methylation and meQTLs in fetal and adult brains from controls and people diagnosed with schizophrenia (SCZ). Jaffe et al. [126] assessed 230,000 CpGs during fetal development. Among these, they identified 6480 differentially methylated regions (DMRs) during the transition from the second fetal trimester to postnatal life. These DMRs mapped to 4557 genes. Furthermore, the genetic risk loci for SCZ, previously identified by the Psychiatric Genetics Consortium (PGC), contained 2903 CpGs that were differentially methylated during the perinatal transition phase. In an independent study on adult brains, 2104 CpGs were differentially methylated between controls and people diagnosed with SCZ in the dorsolateral prefrontal cortices (marked in light green and orange, respectively). Moreover, 97 CpGs corresponded to adult meQTLs but not another set of 40 CpGs that mapped to PGC loci. Interestingly though, Hannon et al. found in an independent study [125] as indicated by the dashed line that 62 out of 104 genome-wide significant PGC loci contained an adult meQTL.

Both studies first sought to investigate the role of CpG methylation and meQTLs during fetal development. Jaffe et al. [126] assessed genome-wide DNA methylation at 230,000 CpGs in DLPFCs collected from 335 individuals ranging in age from the 14th week of gestation to 80 years of age, who were unaffected by psychiatric disease. This approach led to the identification of 6480 statistically significant DMRs that emerged during the transition from the 2nd fetal trimester to postnatal life and that were mapped to 4557 genes (Figure 3). Most of these genes shared a crucial role in brain development and morphogenesis.

**Figure 4:**
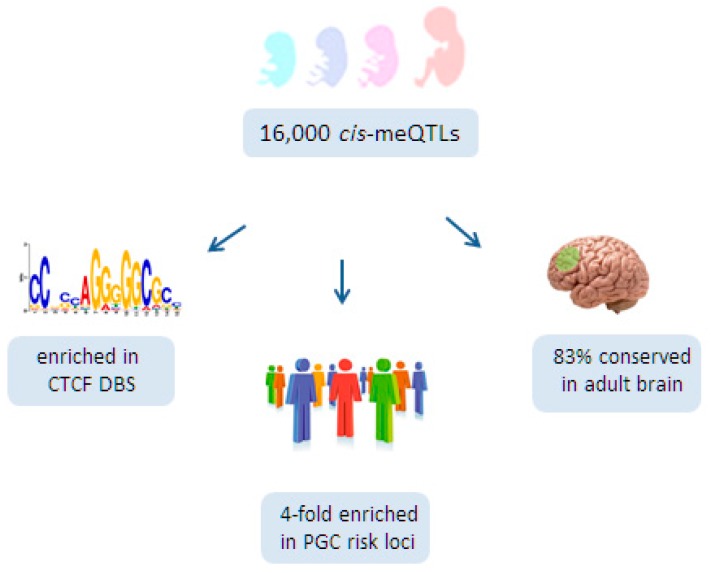
A role for fetal *cis*-meQTLs in schizophrenia. Hannon et al. [125] detected 16,000 *cis*-meQTLs in fetal brains aged 56 to 166 days post-conception. Fetal brain *cis*-meQTLs are strongly enriched in DNA binding sites (DBS) for the transcriptional regulator CTCF. Additionally, fetal brain *cis*-meQTLs are four-fold enriched for genetic risk variants identified previously by the Psychiatric Genetics Consortium (PGC). Lastly, 83% of the fetal *cis*-meQTLs are maintained beyond fetal life in the adult brain.

At the same time, Hannon et al. [125], detected ≈16,000 *cis*-meQTLs within a 1 Mb sliding window in 166 fetal brains ranging from 56 to 166 days post-conception (Figure 4). In agreement with a previous report [124], the effect sizes of these meQTLs were small, with a median change in DNA methylation per allele of ≈7%. Likewise, only a few *trans*-meQTLs (*n* = 5) of smaller effect size were found. 

Functionally, fetal brain meQTLs fulfilled the criteria of regulatory domains [134,138]; they were characterized by the presence of DNase I hypersensitive sites, regulatory histone marks, transcription factor binding sites, and eQTLs. Interestingly, fetal brain meQTLs were strongly enriched in DNA-binding sites for the architectural protein CTCF (Figure 4) that connects higher-order chromatin structure to lineage-specific, but also to aberrant, gene expression [139]. Furthermore, a recent integrated approach for pathways and genes disturbed in SCZ has pointed to a role for CTCF [140]. By connecting genetic variation to genomic function, CTCF may operate as an important organizational factor for fetal meQTLs. 

Extending beyond fetal development, both research teams analyzed next the relationship between CpG methylation, meQTLs, and SCZ, regardless of genetic changes identified by previous GWAS.

Interestingly, Hannon et al. [125] found that 2903 CpGs located in PGC risk loci for SCZ were more likely to show differential methylation during the transition of prenatal to postnatal life than non-SCZ risk loci (Figure 3). Additionally, fetal brain meQTLs were four-fold enriched for genome-wide significant PGC risk variants, suggesting further a developmental role for SCZ risk variants (Figure 4). Consistent with these findings, PGC risk variants are hypothesized to play a role in neurotransmission (glutamatergic-, calcium-, and G-protein coupled receptor signaling), synaptic plasticity, and neurodevelopment. Moreover, 83% of the fetal meQTLs persisted also into adulthood and were detected in at least one of the three brain regions (prefrontal cortex, striatum, and cerebellum) analyzed (Figure 4).

In an independent approach, Jaffe et al. [126] performed additionally a comprehensive meQTL analysis in a large set of adult cortices (191 individuals diagnosed with SCZ and 240 matched controls). The researchers found that 62 out of 104 genome-wide significant PGC loci contained a meQTL within 20 kb of tag SNPs and those in LD (*R*^2^ > 0.6) (Figure 3). While none of these PGC meQTLs was specific to control or disease status, they could still influence SCZ development in response to environmental exposures. 

At the same time, Jaffe et al. identified 2104 CpGs in the adult brain that were differentially methylated between SCZ cases and controls. These CpGs were weakly but significantly enriched in PGC risk loci (40 CpGs out of 2104 CpGs). On the other hand, only 97 diagnosis-associated CpGs corresponded to genome-wide significant meQTLs, suggesting that diagnosis-associated CpGs are distinct from SCZ risk loci-associated meQTL.

Taken together, the key findings from these two pioneering studies are that a substantial fraction of PGC risk loci contain a meQTL (62 out of 104 loci) [126] and that fetal meQTLs, which mostly persist (83%) into the adult brain, are four-fold enriched with PGC risk loci [125]. Therefore, genetic variation directing differential DNA methylation in neurodevelopmental genes may constitute an import risk factor in SCZ. 

## 7. Conclusions and Outlook

Early brain plasticity provides a unique substrate for epigenetic mechanisms in the mediation between a genetic blueprint and adverse environments. Such interactions can trigger sustained epigenomic changes that preserve early life events and thereby contribute to the development of MDD/SCZ. Genome-wide changes in DNA methylation in response to early adversity have been detected in MDD, which is consistent with a major environmental component in this disease. Moreover, genetic variations controlling dynamic DNA methylation in early life are recognized to influence later epigenomic changes in SCZ. This finding is consistent with SCZ’s high genetic load and neurodevelopmental origin and strengthens the concept that epigenetic changes in response to the environment are, at least in part, genetically controlled. At the same time, meQTLs are enriched in GWAS risk variants, supporting their role in SCZ. In both MDD and SCZ, epigenomic changes localize to regions containing genes important to different aspects of early brain development, including neural proliferation, differentiation, and synapse plasticity, among others. 

The genomic distribution of DNA methylation encodes important biological information and is central to ongoing efforts to understand its role in development and disease. DNA methylation analysis technology has rapidly progressed over the past decade [141], whereby the implementation of array hybridization techniques greatly facilitated early genome-scale analysis of DNA methylation. Endonuclease-treated or affinity-enriched DNA methods (used in references [96,98,100,101,102,110]) are particularly well suited for array hybridization. More recently, high density CpG array systems have been broadly used for large clinical samples (used in references [111,112,125,126,132,137]). This technology enables content selection independent of the bias-associated limitations often associated with methylated DNA capture methods and combines comprehensive coverage and high-throughput capabilities. In this regard, the recently introduced MethylationEPIC BeadChip covers more than 850,000 methylation sites, enabling a pan-enhancer and coding region view of the methylome.

While whole genome bisulfite sequencing has the advantage of theoretically capturing all cytosines in the genome at single-nucleotide resolution, it has also a number of significant practical drawbacks as its cost and inefficiency are limiting its broad use despite decreasing sequencing costs. As an alternative, targeted bisulfite sequencing of the dynamic DNA methylome [142] maintains the ability to link cytosine methylation to genetic differences, the single-base resolution, and the analysis of neighboring cytosines, while notably reducing the cost per sample by focusing the sequencing effort on the most informative and relevant regions of the genome. Since no single technique covers at present all aspects, sample numbers and characteristics, as well as the desired accuracy, coverage, and resolution, continue to influence the choice of technique until single-molecule and nanopore sequencing approaches are likely to catalyze the next transformation in high-throughput DNA methylation analysis [143].

At this time, postmortem epigenomic studies on MDD and SCZ have still to overcome a number of limitations to unfold their full potential. For MDD, larger sample sizes are needed to extend the present findings and to assess the possibility of genetic variation in vulnerability to early life adversity. Since our knowledge on epigenomic changes in MDD is still based on the hybridization of DNA recovered from immunoprecipitation to customized tilling arrays, a pan-enhancer and coding region view of the methylome in MDD is urgently looked for. In this respect, postmortem epigenomic studies in SCZ are more advanced compared to those in MDD, both in terms of sample size and comprehensive genome-wide coverage, although the potential relationship between genetically controlled methylation changes and early life events, if at all, needs to be explored in greater depth. 

It is also important to note that accumulating evidence from human, animal, and in vitro studies that indicates that antipsychotic and antidepressant agents can influence the epigenetic machinery and lead to changes in DNA methylation, histone modifications, and possibly micro RNA expression [109,144]. Likewise, the gradually developing but persistent therapeutic effects of antidepressant medications may be achieved in part via epigenetic mechanisms. Although current studies on epigenetic deregulation in SCZ and MDD have accounted for various demographic variables such as age, sex, and post-mortem interval, the effects of medication are still incompletely acknowledged. This failure refers to dosage regimen, combination therapy, and the duration of therapy. Moreover, treatment responses to psychopharmacological therapy depend as well on genetic variation [145]. Therefore, future studies on post-mortem epigenomic changes in major psychosis need to take into account that genotypes, epigenetic mechanisms, and psychopharmacology may interact at multiple layers. 

Given the scarcity of well-documented and suitable brain samples (e.g., from suicide with a history of early life adversity) cross-sectional and longitudinal studies on genome-wide methylation changes in peripheral blood cells have been increasingly adopted for practical reasons [146]. These approaches can deliver biomarkers for disease onset, progression, and therapeutic responses; however, they appear less suitable to elucidate the molecular and cellular processes underlying the actual disease processes in MDD and SCZ. Cell-type specific differences in the response to the environment and the genetic control of DNA methylation are likely to exist between peripheral blood cells and the brain but also within each tissue, particularly in the brain [147]. Challenges from tissue heterogeneity can confound spatiotemporal effects from environmental exposures and from genetic variation, and ultimately, conceal functional causality. For example, the epigenetic programming of *Nr3c1* in response to early life stress in mice shows a high degree of cell-type specificity in the paraventricular hypothalamic nucleus that associates with distinct endocrine and behavioral phenotypes upon renewed stress exposure [106]. In view of the brain’s high specialization, epigenomic changes have to be mapped to cells to function to promote insight into complex disease processes and drugable targets.

Relatedly, SCZ-associated SNPs account for rather small changes in methylation differences, which correspond to 1.3% of average methylation differences in diseased and control prefrontal cortex samples [126] and a 6.7% difference per allele for the average meQTLs [125]. Both studies do not investigate the effects on gene expression in homogenized tissues that are very likely to dilute cell-type specific effects and biologically relevant variability at the level of single cells. Refined biostatistical methods can resolve, at least in part, such limitations by correcting for differences in cell type compositions [148,149]. Moreover, recently developed single-cell assays for genome, epigenome, and transcriptome analysis provide new opportunities to advance cellular resolution in healthy and diseased brains [150,151].

Beyond technical improvements, an intriguing question is whether epigenomic changes in MDD/SCZ are to be expected to translate into gene expression changes in postmortem brain to be classified as functional, and, by inference, disease-relevant. Studies on postmortem brains from suicide completers with and without a history of early life adversity have focused on epigenomic changes in regions associated with gene expression changes in neurodevelopmental pathways. In case these epigenomic changes are established preferentially in early life, they can, but must not, underlie changes in gene expression in adult life. In the absence of longitudinal studies, primary and secondary epigenomic changes remain undistinguishable, as are their potential effects on gene expression.

Similarly, the origin and functional consequences of fetal meQTLs remain presently unanswered. Conceptually, meQTLs map genome-wide DNA methylation levels to genetic variation but do not pinpoint causal variants, a caveat that applies as well to conventional GWAS. Hence, meQTLs can affect DNA methylation at multiple genes that may or may not translate in early and/or adult gene expression changes, irrespective of a causative role. Furthermore, the effects of meQTLs on gene expression may be confined to spatiotemporal time windows or encode gene expression potential that depends on (renewed) neuronal activation to manifest [152]. Taken together, constraints in gene regulation during development and beyond could well explain the low correlation between meQTLs and gene expression levels reported so far. 

In this context, induced pluripotent stem cells (iPSCs) [153] from case and control subjects could provide an interesting opportunity to address part of these questions. iPSCs recapitulate major features of fetal brain cells and can be differentiated into various cell types [154]. Moreover, organoids derived from 3D culture show gene expression profiles [155], epigenomic features [156], and structural self-organization into various regions [157], closely mimicking fetal tissues. The role of non-coding variants of fetal meQTLs in DNA methylation and gene expression can be further assessed within such cellular models by using programmable nucleases (e.g., RNA-guided engineered nucleases derived from the bacterially clustered, regularly interspaced short palindromic repeat (CRISPR-Cas) associated system). These tools enable us to evaluate the effects of fetal meQTLs through high precision genome editing in an early neurodevelopmental context. While offering potential insight into the role of genetically controlled methylation, it has to be kept in mind that DNA methylation in iPSCs is often aberrant and incompletely reset with differentiation [158]. Such failure can critically confound the interpretation of disease-specific epigenetic mechanisms in iPSC derived neural systems and makes the investigation of multiple, independently generated iPSC clones or populations mandatory. 

Overall, recent technical advancements can throw new light on the question of to which degree neuronal epigenomes encode past and present gene expression profiles and how these intersect with genetic risk variants from GWAS. Current findings from MDD/SCZ raise the possibility that neuronal epigenomes trace expression patterns from developmental time windows that are particularly susceptible to environmental or genetic disturbances that may influence vulnerability to disease. If this is the case, timely therapeutic intervention may help to attenuate these processes. The reversibility of epigenetic processes lends wings to this perspective and may offer hope for improving the lives of patients and their families.
